# Apical-Out Human Airway Organoids Modeling SARS-CoV-2 Infection

**DOI:** 10.3390/v15051166

**Published:** 2023-05-14

**Authors:** Man Chun Chiu, Shuxin Zhang, Cun Li, Xiaojuan Liu, Yifei Yu, Jingjing Huang, Zhixin Wan, Xiaoxin Zhu, Jie Zhou

**Affiliations:** 1 Department of Microbiology, Li Ka Shing Faculty of Medicine, School of Clinical Medicine, The University of Hong Kong, Hong Kong, China; mcchiu09@connect.hku.hk (M.C.C.);; 2 State Key Laboratory of Emerging Infectious Diseases, The University of Hong Kong, Hong Kong, China; 3 Centre for Virology, Vaccinology and Therapeutics, Hong Kong Science and Technology Park, Hong Kong, China

**Keywords:** human airway organoids, epithelial polarity, SARS-CoV-2, Omicron variant

## Abstract

The respiratory epithelium, particularly the airway epithelium, is the primary infection site for respiratory pathogens. The apical surface of epithelial cells is constantly exposed to external stimuli including invading pathogens. Efforts have been made to establish organoid cultures to recapitulate the human respiratory tract. However, a robust and simple model with an easily accessible apical surface would benefit respiratory research. Here, we report the generation and characterization of apical-out airway organoids from the long-term expandable lung organoids that we previously established. The apical-out airway organoids morphologically and functionally recapitulated the human airway epithelium at a comparable level to the apical-in airway organoids. Moreover, apical-out airway organoids sustained productive and multicycle replication of SARS-CoV-2, and accurately recapitulated the higher infectivity and replicative fitness of the Omicron variants BA.5 and B.1.1.529 and an ancestral virus. In conclusion, we established a physiologically relevant and convenient apical-out airway organoid model for studying respiratory biology and diseases.

## 1. Introduction

The human respiratory tract, particularly the airway epithelium, is exposed to the external environment, and therefore it is vulnerable to microbial invasion. Respiratory viruses such as influenza A viruses, SARS-CoV, MERS-CoV and the latest SARS-CoV-2 pose major threats to public health globally. The human airway, from the nasal cavity to the terminal bronchioles, is lined with a pseudostratified polarized epithelium composed of four major airway epithelial cell types. Commonly used cell lines, which are homogenous, transformed and/or cancerous cells, cannot simulate the multicellular composition and delicate functionality of the human airway epithelium. The use of alternative models such as primary epithelial cells is hindered by a limited proliferation capacity in vitro since the conventional cell culture approach is unable to sustain the long-term growth of primary epithelial cells. Additional issues involve the limited accessibility of human tissues for establishing the primary culture and the variation in individual tissues. Thus, primary cell culture is not readily available or applicable for research purposes. The past decade has witnessed a major breakthrough in human biology, i.e., the in vitro generation of organoids [1,2]. Organoid culture derived from adult stem cells (ASCs) has been established for most human organs, including the long-term expandable human respiratory organoid culture system established by us [3,4,5]. Lung-tissue-derived lung organoids (LOs) can be long-term passaged and expanded for more than 1 year. Long-term expanding lung organoids contain various proportions of airway and alveolar epithelial cells, including basal cells, ciliated cells, goblet cells, club cells and type 1 and type 2 alveolar epithelial cells (AT1 and AT2) and retain the progenitor cells of both lineages, enabling bi-potential differentiation into the airway or alveolar organoids [5]. Upon proximal differentiation, the resultant airway organoids (AwOs) faithfully simulate the multicellular composition and mucociliary clearance function of the airway epithelium. We have demonstrated that these human airway organoids, in three-dimensional (3D) and two-dimensional (2D) monolayer formats, can be utilized to assess and predict the infectivity of influenza viruses and coronaviruses in humans [3,5].

Applying 3D culture is an essential step in establishing and maintaining ASC-derived organoids. Organoids are embedded in an extracellular matrix (ECM) such as matrigel and overlaid with the medium supplemented with a defined cocktail of niche factors. Organoids, including airway organoids, grown in ECM exhibit an apical-in polarity. Namely, the apical surface of the polarized epithelial cells is enclosed inside the organoid lumen, while the secreted mucus and cellular debris are trapped inside the lumen. In the absence of any intervention, this apical-in polarity is not compatible with the natural scenario of respiratory epithelium exposure to pathogens and the external environment. Normally, the human respiratory epithelium is exposed to microbes and external stimuli via the apical surface. To better simulate the virus–host interaction in the native human airway epithelium, additional manipulations are required in the airway organoids of apical-in polarity, e.g., microinjection into the central lumen, mechanical shearing of the organoids or transformation into a 2D monolayer onto transwell inserts [2,6]. However, these manipulations, e.g., microinjection and mechanical shearing, are either labor-intensive or may induce physiological changes in the organoids. Co et al. reported that the removal of ECM proteins generated enteroids with apical-out polarity [7]. Here, we were prompted to establish apical-out human airway organoids with a readily accessible apical surface, and provide an improved organoid model for studying respiratory infections.

## 2. Materials and Methods

### 2.1. Adult-Stem-Cell-Derived Human Lung Organoids

#### 2.1.1. Establishment and Maintenance of Lung Organoids

This study was approved by the Institutional Review Board of the University of Hong Kong/Hospital Authority Hong Kong West Cluster (UW13-364 and UW21-695). Three lines of human lung organoids were established from surgically resected normal lung tissues according to our previously published protocols [3,5,8]. The lung organoids were 3D-cultured in matrigel overlaid with an expansion (Exp) medium. The lung organoids were passaged every 2–3 weeks or subjected to proximal differentiation when needed.

#### 2.1.2. Proximal Differentiation and Polarity Reversal of Airway Organoids

The lung organoids were cultured in the expansion medium for 7–10 days after passaging, followed by incubation in a proximal differentiation (PD) medium (PneumaCult™-ALI medium + 10 μM Y-27632 + 10 μM DAPT + 100 μg/mL primocin) for generating mature airway organoids in three formats: apical-in, apical-out and 2D monolayer on transwell [3,8].

For generating apical-in airway organoids, lung organoids were maintained within the matrigel overlaid with the PD medium for 14 days.

For generating apical-out airway organoids, lung organoids were recovered from matrigel and then suspension-cultured in PD medium in Nunclon Sphera culture plate (Thermo Scientific, Waltham, MA, USA) for 14 days.

For generating 2D monolayer airway organoids, lung organoids were dissociated into single-cell suspension with 10× TrypLE (Invitrogen, Waltham, MA, USA) digestion, followed by mechanical shearing with a glass Pasteur pipette, and filtering over a 40 μm cell strainer (Falcon, Waltham, MA, USA). The dissociated cells were seeded onto 12-well or 24-well transwell inserts (Corning, Corning, NY, USA), followed by incubation in the expansion medium for 2 days and then the PD medium for 12–14 days.

The bright-field images of the organoids were acquired using a Nikon Eclipse TS100 Inverted Routine Microscope. The videos of the organoids were acquired using a Nikon Eclipse Ti2 Inverted Microscope System. Samples were collected at 14 days post-differentiation for detection of cellular gene expression, immunofluorescence staining or flow cytometry analysis.

### 2.2. Viruses

#### 2.2.1. Preparation of Virus Stock

SARS-CoV-2 isolate HKU-001a (WT, GenBank accession number MT230904), Omicron variants B.1.1.529 (GenBank accession number OM212473) and BA.5 (GISAID accession number EPI_ISL_13777658) were previously reported [9,10,11]. These viruses were propagated in VeroE6/TMPRSS2 cells (JCRB1819) purchased from JCRB Cell Bank, and then quantified by viral gene copy number or titrated with a plaque assay or TCID_50_ as we described previously [5,12,13].

#### 2.2.2. Infection of Human Airway Organoids

The differentiated apical-out airway organoids were incubated with SARS-CoV-2 (WT, B.1.1.529, BA.5) at the multiplicity of infection (MOI) of 1 for 2 h at 37 °C. After incubation, the organoids were washed to remove the unbounded viruses and then cultured in basal medium (Advanced DMEM/F-12 + 1% HEPES + 1% GlutaMAX + 1% Penicillin–Streptomycin). At the indicated time points, samples were harvested for viral quantification, flow cytometry and immunofluorescence staining.

### 2.3. Antibody Treatment

The matrigel-embedded lung organoids were differentiated in PD medium for 1 week, and then treated with 5 μg/mL anti-integrin β1 antibody (Table 1) or control IgG diluted in PD medium for 1 week. The resultant airway organoids were collected for subsequent immunofluorescence staining.

### 2.4. Dextran Permeability Test

The apical-in and apical-out airway organoids were live-stained with Hoechst 33,342 (Thermo Scientific, Waltham, MA, USA) and CellMask Deep Red Plasma Membrane Stain (Invitrogen, Waltham, MA, USA) to visualize the nuclei and cell membrane. Then, the organoids were incubated with 2 mg/mL fluorescein isothiocyanate (FITC)– dextran (10 kDa) (Sigma-Aldrich, Burlington, MA, USA) diluted in PD medium and then live-imaged with an Olympus BX53F Upright Microscope System.

### 2.5. RNA Extraction, RT and qPCR

For detection of the target gene (Table 2) expression level, organoids were lysed and applied to RNA extraction using a MiniBEST Universal RNA Extraction kit (Takara, Kusatsu, Japan), followed by reverse transcription using a Transcriptor First-Strand cDNA Synthesis Kit (Roche, Basel, Switzerland) with oligo(dT) primer, and finally quantitative polymerase chain reaction using the LightCycler 480 SYBR Green I Master Mix (Roche, Basel, Switzerland) and LightCycler 96 instrument (Roche, Basel, Switzerland). The target gene expression levels were normalized to that of the housekeeping gene *GAPDH*.

### 2.6. Immunofluorescence Staining

To visualize the target cellular proteins or viral proteins, organoids were harvested for immunofluorescence staining. Briefly, the organoids were fixed with 4% paraformaldehyde (PFA) for 1 h, permeabilized with 0.5% Triton X-100 for 10–20 min and then blocked with 3% bovine serum albumin (BSA) for 1 h. Then, the organoids were incubated with primary antibodies (Table 1) at 4 °C overnight, followed by secondary antibodies. Nuclei and F-actin were counterstained with DAPI (Invitrogen, Waltham, MA, USA) and Phalloidin-647 (Sigma-Aldrich, Burlington, MA, USA), respectively. Confocal images were acquired with a Carl Zeiss LSM 800 confocal microscope.

### 2.7. Flow Cytometry Analysis

To determine the cellular composition of the organoids, organoids were harvested for flow cytometry analysis. Briefly, the organoids were dissociated into single-cell suspension with 10 mM EDTA (Invitrogen, Waltham, MA, USA) for 1 h at 37 °C, followed by mechanical shearing with a glass Pasteur pipette and filtering over a 40 μm cell strainer (Falcon, Waltham, MA, USA). The dissociated cells were fixed with 4% PFA for 1 h, permeabilized with 0.1% Triton X-100 for 5 min and incubated with primary antibodies (Table 1) that can recognize basal (CK5), ciliated (ACCTUB), goblet (MUC5AC) or club cells (CC10) for 1 h, followed by secondary antibodies.

To determine the infection rate of the organoids, mock-infected and SARS-CoV-2 (WT, B.1.1.529, BA.5)-infected organoids were harvested for flow cytometry analysis with an anti-double-stranded RNA (dsRNA) antibody (Table 1). The immunostained cells were re-suspended in 2% FBS/PBS and analyzed using a BD FACSCanto II system.

### 2.8. Statistical Analysis

Statistical analysis was conducted using GraphPad Prism 9.0. Either Student’s *t*-test or the ANOVA test was used to determine statistical significance as specified in the figure legends. Experiments were repeated in two or three organoid lines, and representation results are shown in the figures. The number of replicates is indicated in the figure legends. * *p* ≤ 0.05, ** *p* ≤ 0.01, *** *p* ≤ 0.001.

## 3. Results

### 3.1. Establishment of Apical-Out Human Airway Organoid Model

#### 3.1.1. Generation of Apical-Out Airway Organoids

**Figure 1:**
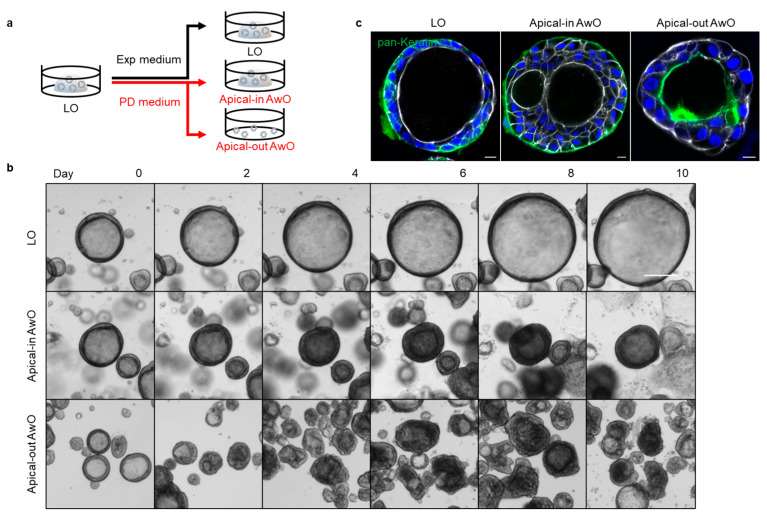
Generation of apical-out airway organoids. (**a**) A schematic graph outlines the expansion of lung organoids (LOs) in expansion (Exp) medium and differentiation of apical-in and apical-out airway organoids (AwOs) in proximal differentiation (PD) medium; (**b**) Bright-field images of the organoids during the expansion or differentiation culture. Scale bar = 200 μm; (**c**) Confocal images of basolateral marker pan-Keratin (green) in lung organoids and airway organoids. Nuclei and actin filaments were counterstained with DAPI (blue) and Phalloidin-647 (white), respectively. Scale bar = 10 μm.

Human lung organoids (LOs) were derived from the lung tissues of three donors who underwent surgical resection due to various medical conditions. To model the natural mode of infections in the human airway by common respiratory viruses, we sought to reverse the apical-in polarity of the airway organoids (AwOs) via removal of the extracellular matrix. To this end, the human lung organoids were split and then suspension-cultured with proximal differentiation (PD) medium in a super-low attachment culture plate, embedded in matrigel (a commonly used approach that generates organoids of apical-in polarity) with the same differentiation medium or embedded in matrigel with the expansion (Exp) medium for 14 days (Figure 1a). Bright-field images were acquired every other day to monitor the morphological changes in the organoids. As shown in Figure 1b, the matrigel-embedded lung organoids in the expansion medium grew gradually with mostly one central lumen. The matrigel-embedded organoids in PD medium did not grow, though they also maintained the central lumen. The organoids suspension-cultured in PD medium became relatively irregular in shape, and some of them lost the central lumen. The organoids only grew when they were cultured in the expansion medium, as shown by the growth kinetics, whereas the organoids switched to the PD medium underwent maturation without discernible growth, regardless of whether they were embedded in matrigel (apical-in airway organoids) or in suspension cultures (apical-out airway organoids) (Figure S1).

Consistent with our previous observation [3,8], abundant ciliated cells were discernible in the differentiated airway organoids in PD medium, both in matrigel and suspension culture. Beating cilia were present on the interior surface of the apical-in airway organoid in matrigel (Video S1) as well as on the exterior surface of the apical-out airway organoid in suspension culture (Video S2), indicating the polarity reversal. Immunofluorescence staining showed the distribution of the basolateral marker pan-Keratin on the exterior basal surface in the lung organoids and apical-in airway organoids versus the interior basal surface in the apical-out airway organoids, which verified the polarity reversal (Figure 1c). These results demonstrate that the removal of the extracellular matrix enabled the generation of apical-out airway organoids.

#### 3.1.2. Characterization of Airway Organoids

**Figure 2:**
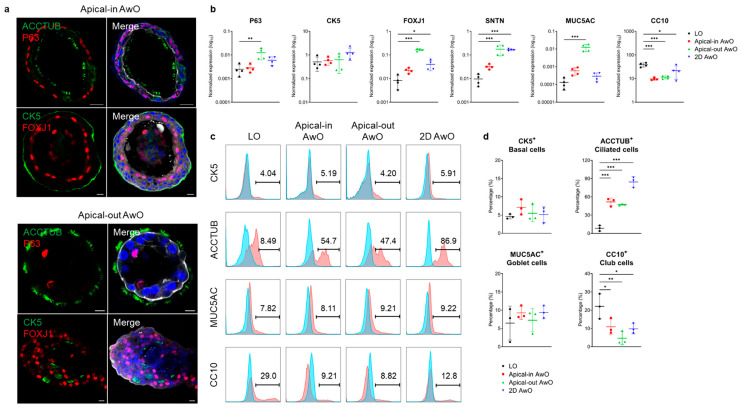
Characterization of airway organoids. (**a**) Confocal images of ciliated cell markers (ACCTUB, green; FOXJ1, red) and basal cell markers (P63, red; CK5, green) in apical-in (top) and apical-out (bottom) airway organoids (AwOs). Nuclei and actin filaments were counterstained with DAPI (blue) and Phalloidin-647 (white), respectively. Scale bar = 10 μm. (**b**) Non-differentiated lung organoids (LOs) and differentiated airway organoids (AwOs) were assessed through qPCR analysis to detect the expression level of marker genes specific for basal (*P63*, *CK5*), ciliated (*FOXJ1*, *SNTN*), goblet (*MUC5AC*) and club (*CC10*) cells. Data represent means ± SD of a representative experiment, *n* = 4. Ordinary one-way ANOVA with Dunnett’s multiple comparison test comparing airway organoids to the lung organoids. * *p* ≤ 0.05, ** *p* ≤ 0.01, *** *p* ≤ 0.001. (**c**,**d**) Non-differentiated lung organoids (LOs) and differentiated airway organoids (AwOs) were assessed through flow cytometry to examine the abundance of CK5+ basal cells, ACCTUB+ ciliated cells, MUC5AC+ goblet cells and CC10+ club cells. Representative histograms are shown in (**c**): red, cells stained with target antibodies; blue, cells stained with isotype controls. Data shown in (**d**) represent means ± SD of a representative experiment in one organoid line, *n* = 3. Ordinary one-way ANOVA with Dunnett’s multiple comparison test comparing airway organoids to the lung organoids.

To test whether the airway organoids of apical-out polarity are well differentiated, we characterized the apical-out airway organoids in parallel with the apical-in airway organoids, 2D airway organoids on transwell inserts and non-differentiated lung organoids. Beating cilia were readily discernible in the apical-in and apical-out airway organoids under the light microscope; immunofluorescence staining of the ciliated cell marker ACCTUB and FOXJ1 verified abundant ciliated cells in both organoids (Figure 2a). The mRNA expression levels of the basal cell marker (*P63*), ciliated cell markers (*FOXJ1*, *SNTN*) and goblet cell marker (*MUC5AC*) were most significantly upregulated in apical-out airway organoids compared to the non-differentiated parental lung organoids (Figure 2b). The club cell marker (*CC10*) was significantly downregulated in all forms of differentiated airway organoids compared to the parental lung organoids (Figure 2b).

The abundance of four major airway epithelial cell types was examined in the organoids through flow cytometry analysis. The percentage of ACCTUB+ ciliated cells increased dramatically after proximal differentiation to approximately 50% in the apical-in and apical-out 3D airway organoids and up to 90% in the 2D airway organoids (Figure 2c,d). The percentages of CK5+ basal cells and MUC5AC+ goblet cells in differentiated airway organoids remained similar to the levels seen in lung organoids, whilst that of CC10+ club cells significantly reduced after differentiation. Overall, both apical-in and apical-out 3D airway organoids phenocopied the cellular composition of the mature airway epithelium to a physiological level. Notably, the 2D airway organoids accommodated a higher proportion of ciliated cells, indicating that transwell culture further facilitated ciliary differentiation.

In addition to the cell-type-specific markers, we also characterized the expression of viral host factors in the organoids. SARS-CoV-2 primarily utilizes ACE2 as the receptor for cell entry. It also relies on cellular proteases for proteolytic activation of spike protein, such as TMPRSS2. The mRNA expression levels of both *ACE2* and *TMPRSS2* significantly increased in the differentiated apical-out airway organoids compared to the non-differentiated parental lung organoids (Figure S2).

The integrity of the epithelial barrier is essential to maintain the homeostasis of the respiratory tract and protect against invading pathogens. To test whether an adequate epithelial barrier was formed in the 3D organoids, we imaged the live apical-in and apical-out airway organoids after treatment with FITC–dextran, a fluorescence-labeled small molecule. Live imaging of the FITC–dextran-treated organoids showed that both apical-in and apical-out airway organoids exhibited an intact epithelial barrier since the fluorescent signal inside the organoid lumen was substantially diminished compared to that surrounding the organoid (Figure S3). In contrast, the EDTA-treated organoids showed enhanced fluorescence intensity within the lumen compared to in the untreated organoids (Figure S3). These results suggest that the apical-in and apical-out airway organoids maintained an intact epithelial barrier and simulated the fundamental attribute of the airway epithelium.

### 3.2. Integrin Beta 1 Controlling the Polarity of Airway Organoids

**Figure 3:**
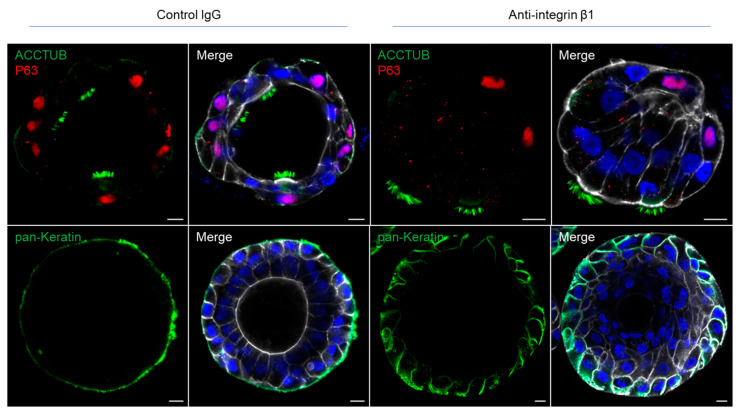
Integrin β1 mediates epithelial polarity of the airway organoids. Matrigel-embedded apical-in airway organoids were treated with function-blocking anti-integrin β1 antibody or control IgG. Confocal images of ciliated cell marker ACCTUB (green) and basal cell marker P63 (red) in control and treated organoids (top). Confocal images of the basolateral marker pan-Keratin (green) in control and treated organoids (bottom). Nuclei and actin filaments were counterstained with DAPI (blue) and Phalloidin-647 (white), respectively. Scale bar = 10 μm.

Cellular polarity is a fundamental feature of epithelial cells. The formation of normal epithelial polarity primarily depends on the interaction between cell surface proteins and the components of the extracellular matrix. One of the candidate proteins involved in the interaction is the integrin, a family of heterodimeric transmembrane glycoproteins composed of a single α subunit and β subunit. Integrin β1 is the major subunit expressed in normal airway epithelial cells [14]. Previous studies have shown that inhibition of integrin β1 by a function-blocking antibody in MDCK spheroids and intestinal organoids induced polarity reversal [7,15]. To assess whether integrin β1 also regulates the polarity of the airway organoids, we examined the polarity of apical-in airway organoids embedded in matrigel after treatment with an anti-integrin β1 antibody or a control IgG for a week. In airway organoids treated by the anti-integrin β1, the normal apical-in polarity of the organoids was disturbed and even partially reverted, as shown by the outward pointing cilia and the redistributed basolateral marker (Figure 3), whereas the organoid treated with isotype control IgG invariably and constantly showed an apical-in polarity. These results suggest that the integrin β1 controlled the polarity of airway organoids.

### 3.3. Differential Replicative Fitness of SARS-CoV-2 Variants in Apical-Out Airway Organoids

The coronavirus disease 2019 (COVID-19) pandemic caused by the severe acute respiratory syndrome coronavirus 2 (SARS-CoV-2) has been a major health and socioeconomic threat since its emergence [16]. SARS-CoV-2 mainly infects epithelial cells in the human respiratory tract. Ciliated cells are the primary target of SARS-CoV-2 [17]. Since its emergence, SARS-CoV-2 has continued to mutate, which has resulted in new variants with enhanced transmissibility. The currently circulating variant of concern, Omicron, emerged in South Africa in late 2021 and became the dominant variant worldwide [18]. Omicron comprises multiple lineages, such as BA.5 [19], and has overtaken the prior circulating variants [20].

**Figure 4:**
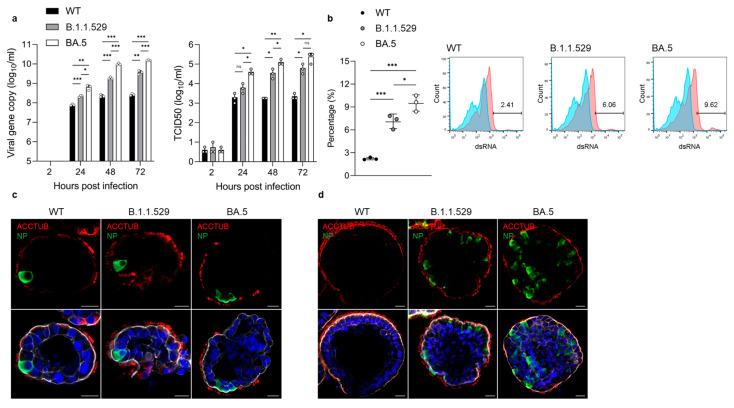
SARS-CoV-2 variants infection in apical-out airway organoids. (**a**) At the indicated hours post-infection (hpi) with SARS-CoV-2 wildtype (WT), Omicron variants B.1.1.529 and BA.5 (1 MOI), culture media were harvested from the infected apical-out airway organoid (AwO) and applied to viral load detection (left) and viral titration (right). Data represent means ± SD of a representative experiment, *n* = 3. Multiple unpaired t-test with multiple comparisons using the Holm–Sidak method was carried out. * *p* ≤ 0.05, ** *p* ≤ 0.01, *** *p* ≤ 0.001. (**b**) At 24 hpi, SARS-CoV-2 WT-, B.1.1.529- and BA.5 (1 MOI)-infected apical-out airway organoids were harvested and assessed using flow cytometry to quantify dsRNA+-virus-infected cells. Data on the left represent means ± SD of a representative experiment, *n* = 3. Ordinary one-way ANOVA with Tukey’s multiple comparison test comparing every other group. Representative histograms are shown in the right: red, infected cells stained with target antibodies; blue, mock-infected cells stained with target antibodies. (**c**,**d**) Confocal images of ciliated cell marker ACCTUB (red) and SARS-CoV-2 nucleoprotein (NP, green) in infected apical-out airway organoids at (**c**) 24 hpi or (**d**) 72 hpi. Nuclei and actin filaments were counterstained with DAPI (blue) and Phalloidin-647 (white), respectively. Scale bar = 20 μm.

To assess whether apical-out airway organoids can model COVID-19 infections, we examined infection and replication of the SARS-CoV-2 Omicron variants B.1.1.529 and BA.5 and an ancestral HKU-001a strain (wildtype, WT) in apical-out airway organoids. The organoids were incubated with the viruses without additional manipulations, which mimicked the nature mode of apical infection in human respiratory epithelium. The apical-out airway organoids sustained productive and multicycle replication of SARS-CoV-2, as shown by an increased viral RNA-dependent RNA polymerase (*RdRp*) gene copy number and infectious titer (Figure 4a). Of note, the Omicron variant BA.5 exhibited the highest replicative fitness in the apical-out airway organoids, having the highest viral load and infectious titer, followed by the B.1.1.529 and then WT virus (Figure 4a). The infection rate as detected through flow cytometry also verified the significantly higher infectivity of the BA.5 and B.1.1.529 variants compared to the WT virus (Figure 4b). Immunofluorescence staining showed more virus-infected cells present in the BA.5-infected organoids compared to the B.1.1.529- and WT-virus-infected organoids (Figure 4c). At 72 h post-infection, there were few viral nucleoprotein (NP)-positive cells in the WT-virus-infected organoids, while significantly more infected cells could be identified in the B.1.1.529-infected organoids and even more in the organoids infected with BA.5 (Figure 4d). Notably, SARS-CoV-2 mainly infected ACCTUB+ ciliated cells in the airway organoid in the earlier stage at 24 h post-infection (Figure 4c). Non-ciliated cells were infected at the later stage at 72 h post-infection (Figure 4d).

Overall, these results demonstrate that the apical-out airway organoids adequately revealed the differential replicative fitness of SARS-CoV-2 variants, which recapitulated the higher transmissibility of Omicron variants, as shown in epidemiological observations.

## 4. Discussion

In this study, we describe the generation of proximal differentiated human airway organoids with “apical-out” polarity. Previously, we used the conventional method of matrigel culture for long-term expansion of non-differentiated lung organoids and generation of differentiated airway organoids. These 3D organoids mostly show “apical-in” polarity, as reported previously [3]. In this study, we demonstrated that the removal of matrigel and suspension culture in a culture plate with minimal cell attachment effectively induced the spontaneous polarity reversal of airway organoids from “apical-in” to “apical-out” polarity (Figure 1).

The proximal differentiation medium enabled the maturation of the apical-out airway organoids (Figure 2 and Figure S3). However, it appeared that the apical-out airway organoids had higher expression levels of ciliated and goblet cell marker genes than the apical-in airway organoids (Figure 2b), probably because matrigel per se could maintain the stemness and proliferative capacity of stem cells and therefore suspension culture further promoted the maturation and differentiation. Nonetheless, flow cytometry data suggest that the apical-in and apical-out airway organoids have similar abundances of ciliated and goblet cells, both of which are comparable to the physiological levels (Figure 2c,d). This discrepancy between mRNA and protein expression levels has been reported previously. Several reasons have been implicated for the mucin proteins, including the unsynchronized level of mRNA and protein in different sampling time points, the higher sensitivity in quantification methods for mRNA than protein and the post-translational regulation of mucin protein synthesis [21].

In particular, the number of ciliated cells, the major airway epithelial cell type, increased significantly from about 10% to 50% (Figure 2d). These ciliated cells have beating cilia on the apical membrane (Videos S1 and S2). Overall, the expression level of cell type markers, cellular composition and barrier function of the apical-out airway organoids were comparable to those of the apical-in airway organoids. Thus, both the apical-in and apical-out differentiated airway organoids morphologically and functionally simulated the human airway epithelium.

The polarity of human airway organoids was also found to be controlled by the integrin proteins, in agreement with previous studies. Blockage of integrin β1 using a function-blocking antibody altered the normal polarity of airway organoids (Figure 3). In the case of apical-out airway organoids, the polarity reversal event induced by matrigel removal and suspension culture may also be mediated by the loss of interactions between the integrin proteins and the extracellular matrix. These findings suggest that integrin proteins may play an important role in the regulation of normal epithelial polarity in the human respiratory epithelium.

While both apical-in and apical-out airways could faithfully simulate the human airway epithelium, the apical-out airway organoid model enables studies of microbial infection in a natural manner. Pathogens such as respiratory viruses can be simply introduced into the culture medium to infect the apical-out organoids, which resembles the mode of natural infection in human respiratory tract. Live imaging of FITC–dextran-treated organoids demonstrated the intact epithelial barrier of the airway organoids (Figure S3), suggesting that the virus inoculum infected the organoids from the exposed outer apical membrane.

During the COVID-19 pandemic, several respiratory organoid models were developed for studying SARS-CoV-2 [22]. Stroulios et al. also reported an apical-out airway organoid model derived from primary human bronchial epithelial cells [23]. Here, we reported the establishment and characterization of an apical-out airway organoid model generated from the long-term expandable and bi-potential lung organoids [5]. This apical-out airway organoid model enables the study of respiratory infection in a physiologically relevant and convenient manner. Indeed, the apical-out airway organoids adequately recapitulated the differential infectivity of emerging SARS-CoV-2 variants and the cellular tropism of SARS-CoV-2 (Figure 4). The rapidly surging infection cases of the Omicron variants suggest that they have competitive advantages over the prior circulating strains. Indeed, the Omicron variants BA.5 and B.1.1.529 have increasing replicative fitness compared to the ancestral strain in the airway epithelial cells (Figure 4a,b). Moreover, it has been reported that SARS-CoV-2 could infect multiple cell types in the human airway epithelium, including ciliated, basal, club, goblet and AT2 cells [24,25]. Among all the target cell types, ciliated cells are the primary target of SARS-CoV-2 in the early stages of COVID-19 [17]. Consistent with those previous findings, SARS-CoV-2 primarily infected ciliated cells in the airway organoids at an early time point and further infected non-ciliated cells at a late time point (Figure 4c,d).

Overall, we have established a robust organoid model for studying respiratory infections and combating pandemics.

## Figures and Tables

**Table 1:** List of Antibodies.

Antibodies/Stains	Suppliers	Catalog No.
Integrin β1	abcam	ab24693
pan-Keratin	abcam	ab8068
CK5	abcam	ab128190
P63	abcam	ab124762
TUBULIN	Sigma-Aldrich	T7941
FOXJ1	Sigma-Aldrich	HPA005714
MUC5AC	abcam	ab3649
CC10	R&D systems	MAB4218
J2 (dsRNA)	Scicons	10010500
NP	in-house [5,12]	n/a

**Table 2:** List of qPCR primers.

Target Genes	Forward Primer	Reverse Primer
*P63*	CAGACTCAATTTAGTGAGCC	CTGCTGGTCCATGCTGTT
*CK5*	GAGGAATGCAGACTCAGTGGA	TAGCTTCCACTGCTACCTCCG
*FOXJ1*	TCGTATGCCACGCTCATCTG	CGGATTGAATTCTGCCAGGT
*SNTN*	GCTGCAAACCCAATTTAGGA	TGCTCATCAAGTTCAGAAAGGA
*MUC5AC*	CCTACAAAGCTGAGGCCTGT	GACCCTCCTCTCAATGGTGC
*CC10*	AGCATCATTAAGCTCATGGAAAAA	GTGGACTCAAAGCATGGCAG
*GAPDH*	GGAGCGAGATCCCTCCAAAAT	GGCTGTTGTCATACTTCTCATGG

## Data Availability

This study includes no data deposited in external repositories.

## Supplementary Materials

The following supporting information can be downloaded at: https://www.mdpi.com/article/10.3390/v15051166/s1. Video S1: beating cilia on the interior apical surface inside the lumen of an apical-in airway organoid. Video S2: beating cilia on the exterior apical surface of an apical-out airway organoid. Figure S1: growth kinetics of organoids. Figure S2: host factors’ expression in organoids. Figure S3: apical-in and apical-out organoids maintain intact epithelial barrier.
