# Supplementary material for: Apical-Out Human Airway Organoids Modeling SARS-CoV-2 Infection
Source: Viruses. 2023 May 14;15(5):1166. doi: 10.3390/v15051166 (PMC10220522; doi:10.3390/v15051166)
Supplement: Supplementary file 1 [file viruses-15-01166-s001.zip › viruses-2319290-supplementary.pdf]

Article

Apical-out Human Airway Organoids Modeling SARS-CoV-2 Infection

Man Chun Chiu [1], Shuxin Zhang [1], Cun Li [1], Xiaojuan Liu [1], Yifei Yu [1], Jingjing Huang [1], Zhixin Wan [1], Xiaoxin Zhu [1] and Jie Zhou [1,2,3,*]

Supplementary Figures

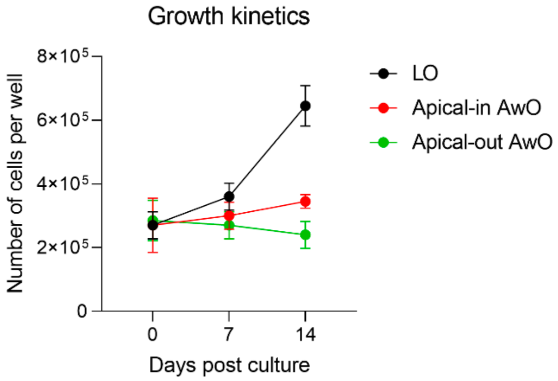

Figure S1. Growth kinetics of organoids. Parental lung organoids (LOs) were cultured in expansion (Exp) medium, or differentiated in proximal differentiation (PD) medium to generate apical-in and apical-out airway organoids (AwOs), respectively. The organoids were dissociated into single cells at the indicated time points and the number of cells in the organoid cultures were counted to monitor the growth of organoids. Data represent means ± SD of a representative experiment, $n = 2$.

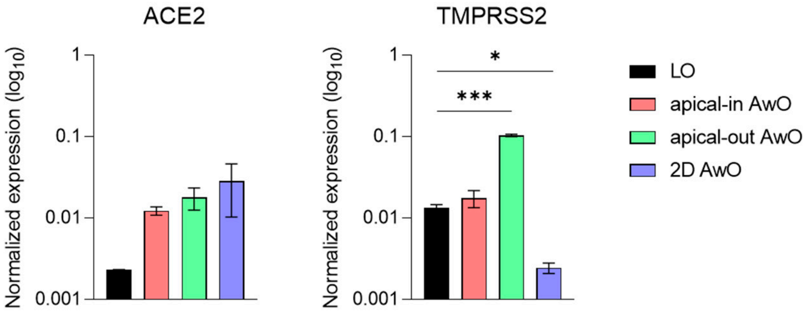

Figure S2. Host factors' expression in organoids. Non-differentiated lung organoids (LOs) and differentiated airway organoids (AwOs) were assessed by qPCR analysis to detect the expression level of *ACE2* and *TMPRSS2*. Data represent means ± SD of a representative experiment, $n = 2$. Ordinary one-way ANOVA with Dunnett's multiple comparison test comparing differentiated airway organoids to the lung organoids. * $p \leq 0.05$, ** $p \leq 0.01$, *** $p \leq 0.001$

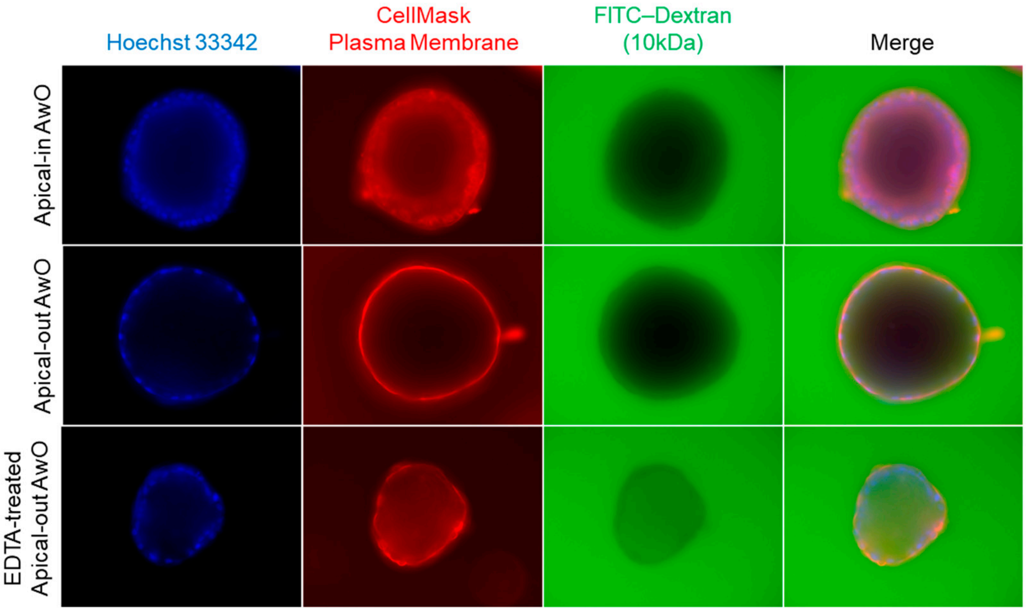

Figure S3. Apical-in and apical-out organoids maintain intact epithelial barrier. Apical-in and apical-out airway organoids (AwOs) were incubated in PD medium with FITC-dextran (green). Representative live images of apical-in and apical-out airway organoids, and EDTA-treated control were shown. Nuclei and cell membrane were stained with Hoechst 33342 (blue) and CellMask plasma membrane (red), respectively.
